# Comparing two fluoride therapies for caries management in young children: study protocol for a randomised clinical trial

**DOI:** 10.1186/s13063-021-05496-y

**Published:** 2021-08-04

**Authors:** Sherry Shiqian Gao, Faith Miaomiao Zheng, Kitty Jieyi Chen, Duangporn Duangthip, Edward Chin Man Lo, Chun Hung Chu

**Affiliations:** Faculty of Dentistry, The University of Hong Kong, Hong Kong SAR, China

**Keywords:** Silver diamine fluoride, Sodium fluoride, Children, Caries, Prevention, Adverse effect

## Abstract

**Background:**

Silver diamine fluoride (SDF) and sodium fluoride (NaF) are widely used for caries management. The objectives of this study are (i) to compare the caries-arresting and caries-preventive effects of SDF and NaF in young children, (ii) to determine children’s and parents’ acceptance of these fluoride therapies and (iii) to investigate the short-term (1 day) and long-term (1 year) adverse effects of these fluoride therapies.

**Methods/design:**

This is a randomised, double-blind, active-controlled clinical trial to be conducted in Hong Kong kindergartens. The study has received approval from the local institutional review board. Written consent will be obtained from the parents/guardians before the study. The study will recruit at least 688 healthy 3-year-old children. This sample size is sufficient for an appropriate statistical analysis. Stratified randomisation will be performed for intervention allocation. The two intervention groups are 38% SDF and 5% NaF varnish applied on six primary upper anterior teeth. At baseline, one trained examiner will perform clinical examinations of the children in the kindergartens. The caries experience and oral hygiene status of each child will be recorded using the decayed, missing (due to caries) and filled primary tooth index and visual plaque index, respectively. Then, an independent operator will apply the assigned fluoride after the dental examinations. The examiner, the children and their parents will be blinded to the intervention allocation. In addition, a research assistant will evaluate the child’s acceptance using interval rating scales for children’s uncooperative behaviour. The examiner will then visit the children the next day to study the short-term potential adverse effects of the fluoride therapies. The same examiner will perform a follow-up examination after 1 year to evaluate the children’s caries experiences, their oral hygiene statuses and the adverse effects of the fluoride. Parental questionnaires will be used to assess parental satisfaction and concerns about the fluoride therapies.

**Discussion:**

This study provides essential information about using SDF in an outreach kindergarten service for caries management from different aspects, which include the caries-arresting and caries-preventive effects, the adverse effects and children’s and parents’ acceptance. The success of the service can help to increase the adoption of SDF to reduce the global burden of early childhood caries.

**Trial registration:**

ClinicalTrials.gov NCT04399369. Registered on May 2020

**Supplementary Information:**

The online version contains supplementary material available at 10.1186/s13063-021-05496-y.

## Background

Early childhood caries (ECC) is defined as the presence of primary teeth affected by carious lesions or white spot lesions, the loss of primary teeth due to caries or the filled primary teeth of a child under 6 years old [1]. The Global Burden of Disease 2017 reported that more than 530 million children had ECC worldwide [2]. The prevalence of ECC was around 70 to 90% in preschool children from most Southeast Asian countries [3]. In Hong Kong, more than half of the 5-year-old children suffered from ECC; more than 90% of the carious teeth were left untreated [4]. The consequences of untreated ECC include not only local pain and infection but also delayed physical growth and development, as well as a diminished quality of life [1]. As with most noncommunicable diseases, the aetiology and progression of ECC are strongly determined based on environmental, economic, social behavioural and societal factors [5]. Studies have reported that tooth brushing behaviours, dietary habits, parental education level and family income were associated with the development of ECC in young children [6, 7]. Moreover, the accessibility to dental care remains inequitable in many places, thus leaving disadvantaged children underserved. Therefore, ECC can be managed through various approaches: improving personal behaviours and habits, working with parents and caregivers, creating a supportive environment, enhancing universal oral health coverage and developing primary care programmes using fluoride therapies, particularly for children from low socioeconomic families.

### Fluoride therapies for managing ECC

Fluoride therapies have been used for managing dental caries for decades. The application of fluoride on tooth surfaces produces calcium fluoride-like globules, which protein phosphate stabilises [8]. They are fairly insoluble in saliva, so they can act as a reservoir of fluoride. When the acidity (pH) in the oral cavity is lowered due to cariogenic challenges, fluoride will be released, and the saturation of phosphate and calcium in plaque fluid will be increased. This function helps to prevent the dissolution of phosphate and calcium ions from enamel and dentine and therefore to enhance the remineralisation of lost minerals [8].

Sodium fluoride (NaF) is one of the most used fluoride products for promoting the remineralisation of dental hard tissues. NaF varnish at 5% (containing 22,600 ppm fluoride) is known to be effective in preventing dental caries. The Cochrane Review reported that it reduced 37% of ECC development in young children [9]. The American Dental Association recommended that fluoride varnish should be applied to high-risk patients for caries prevention at 3- to 6-month intervals [10]. In addition, a systematic review reported that 5% NaF was effective in reversing early enamel (white spot) lesions [11]. Nevertheless, 5% NaF is ineffective in arresting cavitated (dentine) caries in young children [12]. Silver diamine fluoride (SDF) contains silver and fluoride ions. Systematic reviews have found that SDF solution at 38% (containing 44,800 ppm fluoride) was effective in arresting ECC [11, 13]. In addition, a clinical trial reported that SDF was effective for caries prevention in permanent teeth [14]. SDF could exert a preventive effect on the entire primary dentition when applied to carious anterior primary teeth [12]. However, SDF has an unpleasant taste, and it might cause permanent black staining on carious lesions [13]. Both fluoride therapies may be considered for use in young children for preventing and arresting ECC. However, no well-designed clinical study has investigated their effectiveness, children’s and patients’ acceptance of it and its adverse effects when used for caries management.

### Objectives

The objectives of this study are (i) to compare the caries-arresting and caries-preventive effects of SDF and NaF for young children, (ii) to determine children’s and parents’ acceptance of these fluoride therapies and (iii) to investigate the short-term (1 day) and long-term (1 year) adverse effects of these fluoride therapies.

## Methods/design

### Trial design

**Fig. 1:**
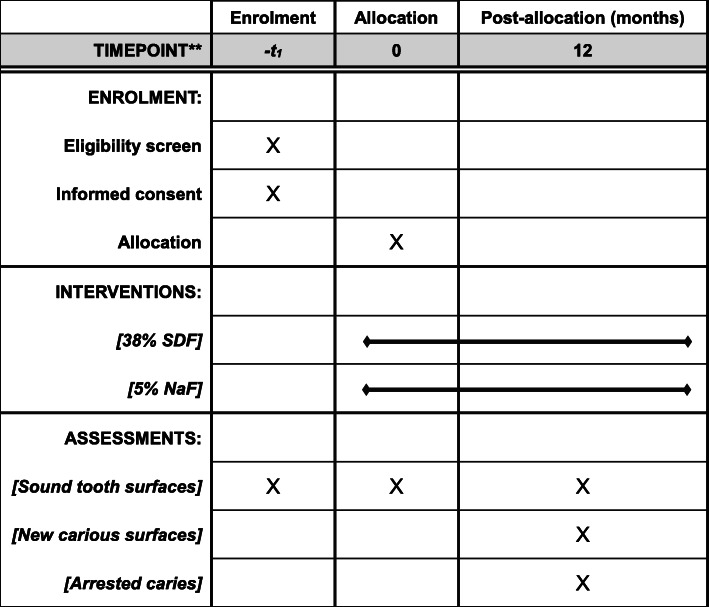
The schedule of enrolment, interventions and assessments

This is an exploratory, randomised, double-blind, active-controlled clinical trial. The design and report of this protocol follow the Standard Protocol Items: Recommendations for Interventional Trials statement (Additional file 1) [15]. The schedule of this study is shown in Fig. 1.

### Setting

This trial will be conducted in kindergartens in Hong Kong. The objectives and procedures of this study will be explained to the kindergarten headmasters. After their confirmation is received, an invitation letter and informed consent form will be sent to the parents of eligible children (Additional file 2). The written consent will be collected from the parents before the trial takes place.

### Participants

All first-year children from the participating kindergartens will be invited to take part in the study. The inclusion criteria will be children who are aged 3 to 4 years old and those who are generally healthy, without taking into account whether they have caries experience or not. Children will be excluded from this study if they are uncooperative with dental procedures; suffer from major systemic diseases; receive long-term medications; have abnormal dentition, such as amelogenesis imperfect; or have tooth anomalies, such as double teeth.

### Baseline and follow-up dental examinations

Baseline and follow-up dental examinations will be conducted in an outreach setting in the participating kindergartens. World Health Organization Community Periodontal Index (CPI) probes (405/WHO probe, Otto Leibinger, Mühlheim, Germany) and light-emitting diode intra-oral illuminations attached to disposable dental mirrors (MirrorLite, Kudos Crown Limited, Hong Kong, China) will be used in the examinations.

A trained dentist will perform dental examinations using visual inspection. No radiography will be taken. The six upper anterior teeth of each child will be studied carefully. The child’s caries status will be evaluated after the tooth surfaces are cleaned and dried with a gauze sponge. All of the surfaces of the six upper anterior teeth will be diagnosed as sound or carious (active or arrested). Surfaces that are free of caries will be recorded as sound. Surfaces with cavitated lesions will be deemed carious. The carious lesions will be explored gently in the lesion’s centre using a CPI probe. If a carious lesion is soft upon gentle probing, it will be diagnosed as active. Meanwhile, if a carious lesion is hard upon gentle probing, it will be diagnosed as arrested [12, 14].

Apart from the six upper anterior teeth, the child’s caries experience at the tooth level will be assessed using the decayed, missing (due to caries) and filled primary tooth (dmft) index. Other dentition problems, such as hyper-mobility, tooth discoloration, abscess and fistula, will be recorded as well. The visible plaque index (VPI) will be used to measure the child’s oral hygiene status [16]. The presence of visible plaque on the buccal and lingual surfaces of six index teeth (55, 51, 63, 71, 75 and 83) will be examined. A single examiner will conduct the baseline and 12-month follow-up dental examinations using the same diagnostic criteria, equipment and procedure. Duplicate tests in 10% of the children regarding caries diagnosis will be conducted at both stages of the study to assess the intra-examiner reliability. To promote the follow-up rate, the research team will keep a good communication with the participated kindergartens; the kindergartens will help to organise the children for a 12-month follow-up examination. In addition, if the children are absent at the follow-up visit, another visit will be conducted within the same week to make up those absent participants.

### Intervention

The two intervention groups are the 38% SDF solution group (Advantage Arrest, Elevate Oral Care, FL, USA) and the 5% NaF varnish group (Duraphat Varnish, Colgate-Palmolive, NY, USA). No placebo-controlled group will be used in this study due to ethical concerns. An independent operator will use micro-brushes (MICROBRUSH, Grafton, WI, USA) to apply the fluoride therapies according to the assigned intervention group on all surfaces of the six upper anterior teeth at baseline. The children will not be allowed to drink or eat for half an hour after the fluoride therapies. Because a dentist is completing this one-off intervention, no special criteria for modifying or discontinuing the intervention, improving the adherence and monitoring the adherence will be issued. Implementing fluoride therapies will not require any alterations to the children’s usual dental care.

### Randomisation, intervention allocation and allocation concealment

This study will use the stratified randomisation method for intervention allocation. The allocation ratio will be 1:1. The enrolled children will first be classified into two strata: (i) low caries risk—having no caries experience (dmft = 0), or (ii) high caries risk—having caries experience (dmft > 0). Children in each stratum will be randomly allocated to two intervention groups using a computer-generated random number with a block size of eight. A research assistant will keep the random number sheet. The allocation sequence will be generated by a separate statistician and sealed in opaque envelopes.

### Blinding

The examiner, the participating children and their parents will not know the intervention allocation throughout the whole study. The blinding will be permissible only when the parents request their children’s treatment histories. The research assistant working with the intervention allocation will disclose the treatment group to the parents. Then, these children will be excluded from this trial.

### Outcome measure

#### Caries-preventive and caries-arresting effect

The caries-preventive effect will be determined based on the number of carious surfaces developed in the six anterior upper teeth at the 12-month follow-up. The caries-arresting effect will be determined based on the number of arrested carious surfaces in the six anterior upper teeth at the 12-month follow-up.

#### Adverse effect

An independent examiner will pay another visit to each kindergarten 1 day after the baseline dental examination and fluoride therapy. The presence of any short-term adverse effects in the hard and soft tissues in the oral cavity will be recorded. At the 12-month follow-up, the examiner will determine the presence of any long-term adverse effects. The expected adverse effects include but not limited to gum irritation, gum swelling, gum bleaching and sound tooth surface discolouration. In addition, the investigator’s contact will be left with the parents. If the parents have any concern or notice adverse effects, they can reach the investigators at their own convenience. Follow-ups will be provided on a case-by-case base.

#### Children’s and parents’ acceptance

At baseline, an independent research assistant will observe the child’s reaction during the dental examination and fluoride therapy. The child’s acceptance of the dental procedures will be measured using interval rating scales for children’s uncooperative behaviour [17]. Their behaviour will be classified as follows:

- 0—Total cooperation, best possible working conditions, no crying or physical protest
- 1—Mild, soft verbal protest or (quiet) crying as a signal of discomfort, but does not obstruct progress; appropriate behaviour for procedure
- 2—Protest more prominent; both crying and hand signals; may move hand around, thus making it hard to administer treatment; protest more distracting and troublesome. However, the child still complies with the request to cooperate
- 3—Protest presents a real problem for the dentist; complies with demands reluctantly, requiring extra effort by the dentist; body movement
- 4—Protest disrupts procedure, requires that all of the dentist’s attention be directed towards the child’s behaviour; compliance eventually achieved after considerable effort by the dentist, but without much actual physical restraint; (may require holding child’s hands, or the like, to start) more prominent body movement
- 5—General protest: no compliance or cooperation; physical restraint is required

It is noteworthy that the children classified as level 5 will be excluded from this study.

A parental questionnaire asking about the parents’ satisfaction levels and concerns about the fluoride therapies will be distributed at the 12-month follow-up to assess parents’ acceptance of the fluoride therapies.

#### Effect modification

Children’s oral hygiene behaviours (e.g. tooth brushing), use of fluoride products (e.g. toothpaste), diet behaviours (e.g. bottle feeding and snacking habits), dental visit behaviours and demographic backgrounds (e.g. parental educational level and monthly family incomes) will be collected through a parental questionnaire administered at baseline.

### Sample size calculation

The data utilised for the sample size calculation is from a previous study conducted in Hong Kong [12]. The mean number of decayed teeth of 3-year-old children was 5. The mean numbers of newly developed carious surfaces in SDF and NaF groups were 0.47 (SD 0.87) and 0.70 (SD 0.84), respectively. The difference in the preventive effects between the two groups was 33%. When the statistical power was set at 0.9 and the type I error was set at 5%, 292 children were required in each group. With the dropout rate estimated at 15%, 688 children should be enrolled at baseline, with 344 children in each group.

### Data analysis

An experienced statistician will oversee the data handling procedures. Two research assistants will enter the data into a Microsoft Excel file independently (double entry). The two datasets will be compared to identify errors. This study will adopt an intention-to-treat analysis. If the quantities of children who withdraw from the study significantly differ between the two intervention groups at the 12-month follow-up, a per-protocol analysis will be conducted. In this case, only the children who have completed the entire trial will be counted in the results. The statistical software products of SPSS for Windows (IBM Corporation, USA) and SAS for Windows (SAS Institute Inc., USA) will be used for the data analyses. The intra-examiner reliability of caries diagnoses will be assessed using Cohen’s kappa statistics at baseline and the 12-month follow-up. The results at both the subject level and the tooth surface level will be analysed. The statistical significance level will be set at 0.05 for two-sided tests.

Chi-square tests will be performed to study the between-group differences in the proportion of children with new caries development (subject level) and in the proportion of tooth surfaces with new cavitations (tooth surface level). *T*-tests will be conducted to study the between-group differences in the mean number of newly developed carious surfaces, the number of arrested carious surfaces, the dmft index and the numbers of non-vital teeth and hyper-mobile teeth at the 12-month follow-up. Descriptive analysis (proportion) will be used to study the adverse effects as well as the children’s and parents’ acceptance of the fluoride therapies. In addition, this study will explore whether patient characteristics affect the treatment effects (effect modification). Independent variables, such as demographic background, dental hygiene practices and other clinical parameters that may modify the outcome variables, will be studied. Because the outcome variables may not be normally distributed, the Poisson model or negative binomial regression model will be considered to investigate the effect modification [18].

### Ethical consideration

Ethical approval has been obtained from the Institutional Review Board of the University of Hong Kong/Hospital Authority Hong Kong West Cluster (UW20-397). A written consent form will be collected from the parents or guardians of each child. All of the participating children have the right to withdraw from this trial at any time for any reason by informing the investigators of their children to do so. Withdrawal from this trial will not influence the children’s right to receive other dental services from the Faculty of Dentistry of The University of Hong Kong. If the participants are harmed by taking part in this study, there are no special compensation arrangements; this information is included in the consent form. Because the clinical procedures performed in this study will be simple and non-invasive, this study will cause minimal risk to the participants. Moreover, professional training sessions will be provided to the field investigators to further minimise the risk. If one life-threatening case appears, or if more than 30% of the children present severe systemic side effects, the study will be stopped. All of the data and related information will be kept in a personal computer confidentially. Only the investigators will have the authority to assess the dataset.

## Discussion

This is a randomised, double-blind, active-controlled clinical trial to study 38% SDF solution and 5% NaF varnish for caries management in young children. These two fluoride therapies are commonly used in outreach-based dental service programmes. We aim to investigate and compare these two fluoride therapies in terms of their caries-arresting effects, their caries-preventive effects, their adverse effects and the children’s and parents’ acceptance of them. The results of this trial can help researchers and clinicians to understand the pros and cons of using 38% SDF and 5% NaF in primary dental care programmes. Dental professionals can adopt the appropriate protocol when developing a new outreach-based caries management project to reduce the global burden of ECC.

NaF varnish at 5% is widely used as a standard of care for preventing ECC, but it is generally considered to be ineffective in arresting ECC [12]. The SDF solution is often used for arresting dental caries, but its effectiveness in preventing ECC has not been studied thoroughly. This clinical trial compares both the caries-preventive and the caries-arresting effect between these two fluoride therapies to comprehend their effectiveness in managing ECC. If 38% SDF is superior to 5% NaF for caries prevention, the standard of care for ECC prevention will be changed. SDF use may be more desirable in managing ECC because both caries-preventive and caries-arresting effects can be achieved through a single SDF application.

This study will explore adverse effects after the fluoride therapies. Both short-term (1 day) and long-term (1 year) adverse effects will be assessed. The safety of NaF varnish has been well proved [19]. However, literatures reporting adverse effects of SDF are few. One previous study reported that gingival irritation (gum pain, swelling and bleaching) was presented in around 5% of the participants after applying SDF on the carious teeth [20]. However, those results were based on parental reports. Moreover, there is no study investigating the adverse effects of SDF when used in caries prevention. In this study, we will send one independent dentist to the kindergartens 1 day after the baseline examination to assess the short-term adverse effects after the fluoride therapies. In addition, the same independent dentist will assess the long-term adverse effects at the 12-month follow-up. Therefore, this study will provide essential information on the adverse effects of the two fluoride therapies.

Patients accept NaF when it is used for caries management [19]. Compared with 5% NaF varnish, 38% SDF may be more difficult for children and their parents to accept. The SDF solution has a metallic taste, which causes unpleasant feelings for patients, especially young children. Therefore, the child’s behaviour during the dental examination and fluoride therapy will be observed in this study. Also, SDF will cause permanent black staining on carious lesions. Some parents were dissatisfied with the appearance of their children’s teeth after SDF applications [20, 21]. Although black staining caused by SDF treatment is always emphasised, it is noteworthy that successfully arrested carious lesion, no matter after SDF or NaF treatment, will generally turn to a dark colour. Other clinical trials did not report the colour change had caused any bias on the blinding issues [12, 14]. Although SDF will not stain sound enamel, it may discolour the plaque or salivary pellicle on the tooth surface. This can affect parents’ acceptance of SDF for caries prevention. Therefore, we will study parents’ acceptance and concerns about the fluoride therapies applied for caries prevention.

The ECC prevalence percentages were 22% and 55% in 3-year-old and 5-year-old Hong Kong children, respectively [4, 6]. The prevalence of ECC increased significantly during the children’s kindergarten lives, and their upper anterior teeth were the most affected teeth [6]. Therefore, this study involved recruiting children who were in their first year of kindergarten and involved treating their six upper anterior teeth. Because this trial is based on an outreach setting, radiography will not be used for caries diagnosis. The examiner will use visual and tactile inspection using CPI probes to detect the presence and statuses of carious lesions. Other clinical studies have demonstrated that this methodology is practical and reliable for assessing dental caries [12, 22].

## Trial status

This clinical trial was registered in ClinicalTrials.gov (U.S.) under the registration number of NCT04399369 in May 2020. This protocol is version 1 developed on 22 July 2020. The recruitment for participation started in September 2020. The recruitment completed in January 2021. The 12-month follow-up is anticipated to be conducted between September 2021 and January 2022.

## Supplementary Information

**Additional file 1.** The Standard Protocol Items: Recommendations for Interventional Trials statement.

**Additional file 2.** An invitation letter and informed consent form.

## Data Availability

The results of the dental examination of each participating child will be shared with the parents via a report. The results of the entire study will be shared in academia via publications and presentations. The datasets generated in this trial will be available from the primary investigator on via a reasonable request.
